# Modeling human enterovirus A71 infection using an intestinal microphysiological system

**DOI:** 10.1128/jvi.00250-26

**Published:** 2026-04-21

**Authors:** Hiroki Futatsusako, Sayaka Deguchi, Kaori Kosugi, Rina Hashimoto, Noriyo Nagata, Tadaki Suzuki, Takuya Yamamoto, Kazuo Takayama

**Affiliations:** 1 Department of Synthetic Human Body System, Medical Research Laboratory, Institute of Integrated Research, Institute of Science Tokyo 13290, Tokyo, Japan; 2 Department of Human Health Sciences, Graduate School of Medicine, Kyoto University 12918, Kyoto, Japan; 3 Department of Infectious Disease Pathology, National Institute of Infectious Diseases, Japan Institute for Health Security 739298 , Tokyo, Japan; 4 Department of Infectious Disease Pathobiology, Graduate School of Medicine, Chiba University 12737, Chiba, Japan; 5 Center for iPS Cell Research and Application (CiRA), Kyoto University 12918, Kyoto, Japan; 6 Institute for the Advanced Study of Human Biology (WPI-ASHBi), Kyoto University, Kyoto, Japan; 7 Medical-risk Avoidance based on iPS Cells Team, RIKEN Center for Advanced Intelligence Project, Kyoto, Japan; University of Minnesota Twin Cities, Minneapolis, Minnesota, USA

**Keywords:** pluripotent stem cell, hand, foot, and mouth disease, embryonic stem cells, organ-on-a-chip, gut-on-a-chip

## Abstract

Enterovirus A71 (EV-A71), a causative virus of hand, foot, and mouth disease, primarily infects and replicates in the intestine and, in severe cases, spreads to the central nervous system, leading to neurological complications. Therefore, suppressing viral replication in the intestine is important to prevent severe complications. However, the intestinal pathophysiological changes in EV-A71-infected patients remain poorly understood. In this study, we aimed to examine the intestinal response to EV-A71 infection using the intestinal microphysiological system (MPS) we previously developed using human pluripotent stem cells and microfluidic devices. The viral titers were detectable in the cell culture supernatant of the intestinal MPS for 14 days after the viral infection. Despite this, EV-A71 infection did not induce significant morphological changes in the intestinal MPS or alter the expression of epithelial cell markers, suggesting that the virus can infect the intestinal MPS without causing intestinal epithelial damage. In addition, we found that the secretion of interferons (IFNs) in the cell culture supernatant was not increased by viral infection. Interestingly, treatment with recombinant IFNs increased the expression of innate immune response-related genes and reduced viral mRNA levels. A strong association was observed between EV-A71 infection and IFN signaling in the intestinal MPS. We believe that the intestinal MPS would be a valuable platform for studying EV-A71 infection and evaluating antiviral strategies.

**IMPORTANCE:**

Enterovirus A71 (EV-A71), a major cause of hand, foot, and mouth disease, primarily replicates in the intestine and can spread to the central nervous system, causing severe neurological complications. Suppressing intestinal replication is therefore critical, yet the intestinal pathophysiology of EV-A71 remains poorly understood. Here, we examined EV-A71 infection using a human pluripotent stem cell-derived intestinal microphysiological system (MPS). Viral titers were detectable in the culture supernatant for 14 days. However, EV-A71 did not induce significant morphological changes or alter epithelial marker expression, indicating persistent infection without intestinal damage. Additionally, EV-A71 infection did not enhance interferon (IFN) secretion. Treatment with recombinant IFNs increased innate immune gene expression and reduced viral mRNA, demonstrating the key role of IFN signaling in restricting infection. These findings suggest that the intestinal MPS would be a useful platform for studying EV-A71 infection and antiviral strategies.

## INTRODUCTION

The genus *Enterovirus* comprises positive-sense single-strand RNA viruses, including enterovirus (EV) species or rhinovirus (RV) species. Among them, poliovirus is the most extensively studied, and global eradication efforts have eliminated most wild strains. Consequently, concerns regarding the endemicity of non-polio enteroviruses have been increasing. Among these, EV-A71 is of particular concern because of its neurotropism (1). EV-A71 typically causes mild illness, such as hand, foot, and mouth disease (HFMD) or common cold; however, in severe cases, it can lead to neurological complications, such as aseptic meningitis and encephalitis.

EV-A71 is predominantly transmitted via the fecal-oral route and primarily infects the intestine. After replicating in the intestine, the virus spreads to other organs. To reduce the risk of severe disease, it is important to inhibit viral replication in the intestine before dissemination to the central nervous system (CNS). However, the intestinal pathophysiology mediated by EV-A71 infection remains poorly understood, and there are no approved drugs against EV-A71 infection.

Rhabdomyosarcoma (RD) cells, HT-29 cells, and Vero cells are widely used in EV-A71 research. However, RD cells are derived from a human rhabdomyosarcoma, HT-29 cells from human colorectal adenocarcinoma, and Vero cells from kidney epithelial cells of an African green monkey. Therefore, they differ from normal human intestinal tissues, making it difficult to recapitulate intestinal responses to EV-A71 infection. Intestinal organoids generated from human adult stem cells have also been used for EV-A71 research (2). While they recapitulate the functions of normal human intestinal epithelial cells, they lack non-epithelial cell types, such as intestinal fibroblasts, which makes it difficult to model the complex responses of EV-A71-infected intestine. Thus, there is a need to develop human models that can recapitulate intestinal responses to EV-A71 infection.

A microphysiological system (MPS) is an *in vitro* model that cultures cells within a microfluidic device. Because physiologically relevant environmental stimuli, such as blood flow-driven shear stress, can be reproduced in the device, this system is expected to recapitulate the complex functions of a human organ. Intestinal MPS has been applied to the study of several viral infections (3). We recently established the intestinal MPS by differentiating human pluripotent stem cells within microfluidic devices (4). This system comprises intestinal epithelial cell types, including enterocytes, goblet cells, enteroendocrine cells, Paneth cells, and intestinal fibroblasts. Within this system, a multilayered mucosal structure, consisting of an intestinal epithelial layer with apico-basal polarity and an aligned stromal layer, was organized. This intestinal MPS is expected to accurately recapitulate the pathophysiology of viral infection. In this study, we aimed to recapitulate the intestinal responses to EV-A71 infection by infecting the intestinal MPS with the virus.

## RESULTS

### EV-A71 infection using the intestinal MPS

**Fig 1:**
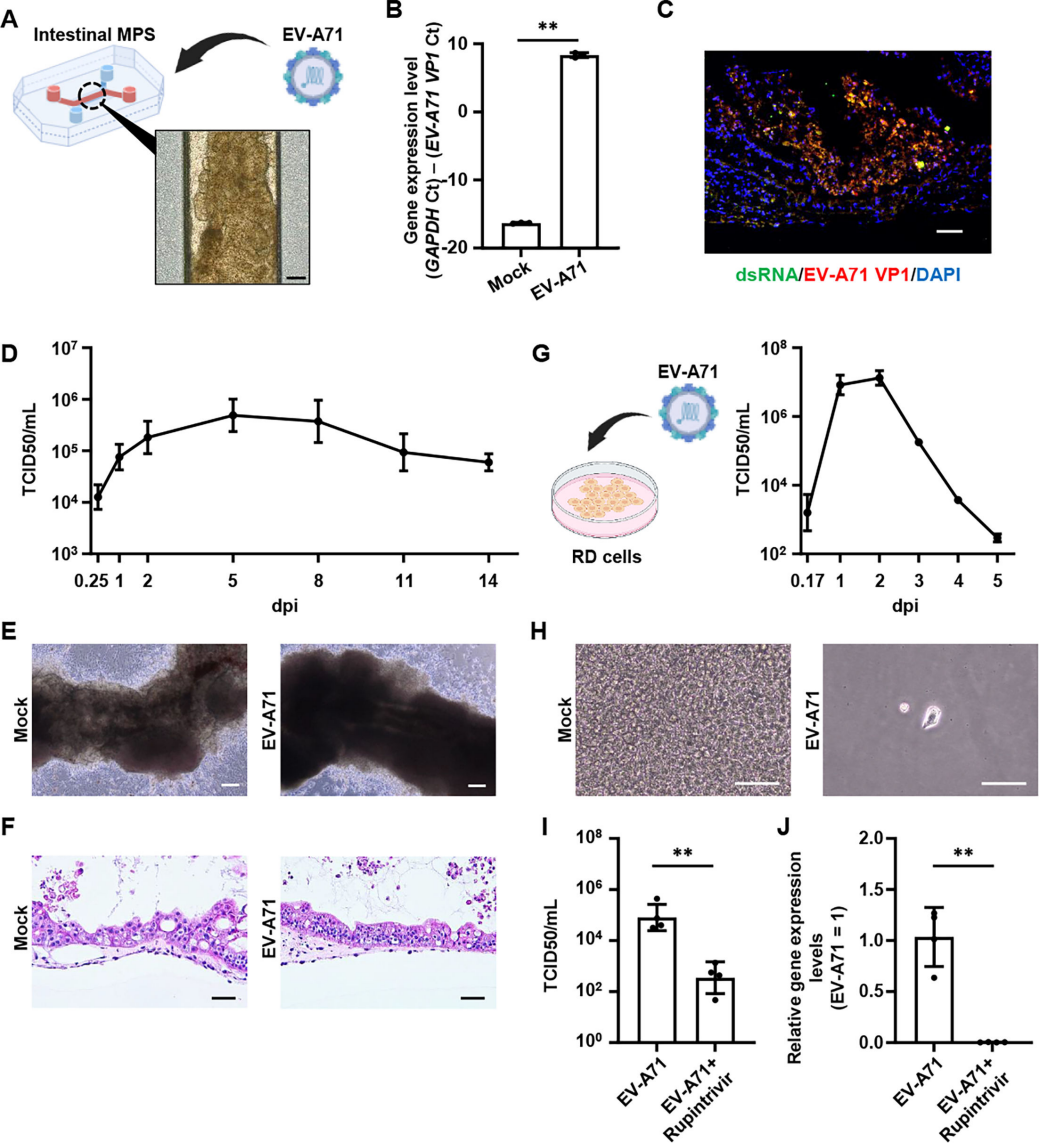
EV-A71 infection using the intestinal MPS. (**A**) The intestinal MPS was infected with EV-A71 at 0.142 TCID_50_/cell. The scale bar represents 200 μm. (**B**) The expression of *EV-A71 VP1* in the intestinal MPS at 7 days post-infection (dpi) was measured by RT-qPCR. Statistical significance was determined using an unpaired two-tailed Student’s *t*-test (**P < 0.01). Data are represented as mean ± SD (*n* = 3). (**C**) Immunofluorescent staining of double-stranded RNA (dsRNA) (green) and EV-A71 VP1 (red) in the intestinal MPS at 5 dpi. Nuclei were counterstained with DAPI (blue). Scale bar represents 50 μm. (**D**) Viral titers in the cell culture supernatant of the intestinal MPS were measured by TCID_50_ assay. Data are shown as mean ± SD (*n* = 4). (**E**) Phase-contrast images of the intestinal MPS at 14 dpi. Scale bars represent 200 μm. (**F**) Hematoxylin and eosin-stained images of the intestinal MPS at 4 dpi. Scale bars represent 50 µm. (**G and H**) RD cells were infected with EV-A71 at an MOI of 0.1. (**G**) Viral titers in the cell culture supernatant of the RD cells were measured by TCID_50_ assay. Data are shown as mean ± SD (*n* = 3). (**H**) Phase-contrast images of RD cells at 4 dpi. Scale bars represent 100 μm. (**I and J**) The intestinal MPS was infected with EV-A71 in the presence or absence of 1 µM rupintrivir. (**I**) Viral titers in the cell culture supernatant of the intestinal MPS at 2 dpi were measured by TCID_50_ assay. Statistical significance was determined using an unpaired two-tailed Student’s *t*-test (**P < 0.01). Data are shown as mean ± SD (*n* = 4). (**J**) The expression of *EV-A71 VP1* in the intestinal MPS in the presence or absence of 1 µM rupintrivir at 7 dpi was measured by RT-qPCR. Statistical significance was determined using an unpaired two-tailed Student’s *t*-test (**P < 0.01). Data are represented as mean ± SD (*n* = 4).

The intestinal MPS was established by differentiating intestinal cells from human ES cells within a microfluidic device. Villin, an intestinal epithelial cell marker, and MUC2, a goblet cell marker, were highly expressed in the intestinal MPS (Fig. S1A). To examine the expression of scavenger receptor class B member 2 (SCARB2), a receptor for EV-A71, immunostaining and capillary-based immunoassay were performed (Fig. S1B through D and S2). Protein level of SCARB2 in the intestinal MPS was comparable to those in RD cells, a widely used cell line for EV-A71 research, and in human colon. Thus, we proceeded to infect the intestinal MPS with EV-A71 (Fig. 1A). Both RT-qPCR and immunostaining demonstrated the presence of viral mRNA and protein in EV-A71-infected intestinal MPS (Fig. 1B and C).

It has been reported that viral RNA was detected in approximately 94% of stool samples from EV-A71-infected patients even 2 weeks after symptom onset (5). We next examined whether EV-A71 infection lasting 2 weeks could be recapitulated in the intestinal MPS. Viral titers in the culture supernatant of EV-A71-infected intestinal MPS remained detectable for 14 days (Fig. 1D). No morphological changes were observed in the intestinal MPS at 14 days post-infection (dpi) (Fig. 1E and F). EV-A71 infection did not affect the viability of the intestinal MPS (Fig. S1E). These results indicate that the intestinal MPS is capable of recapitulating EV-A71 infection in the intestine over a 2-week period. Additionally, RD cells were infected with EV-A71 for comparison with the intestinal MPS. High levels of viral titers were detected in the culture supernatant of EV-A71-infected RD cells at 2 dpi, followed by a gradual decline thereafter (Fig. 1G). EV-A71 infection induced cytopathic effect, and most RD cells were removed following medium change by 4 dpi (Fig. 1H). These results suggest that RD cells do not sustain long-term infection by EV-A71.

Rupintrivir, a 3C protease inhibitor of RV, is known to exhibit antiviral activities against EV-A71 (6). Intestinal MPS was used to evaluate the inhibitory effect of rupintrivir against EV-A71 infection. The intestinal MPS was infected with EV-A71 and treated with rupintrivir. Both viral titers in the culture supernatant (Fig. 1I) and viral mRNA (Fig. 1J) were significantly decreased by rupintrivir treatment. These results suggest that the intestinal MPS is useful for examining the anti-EV-A71 activity of candidate drugs.

### Innate immune responses to EV-A71 infection in the intestinal MPS

**Fig 2:**
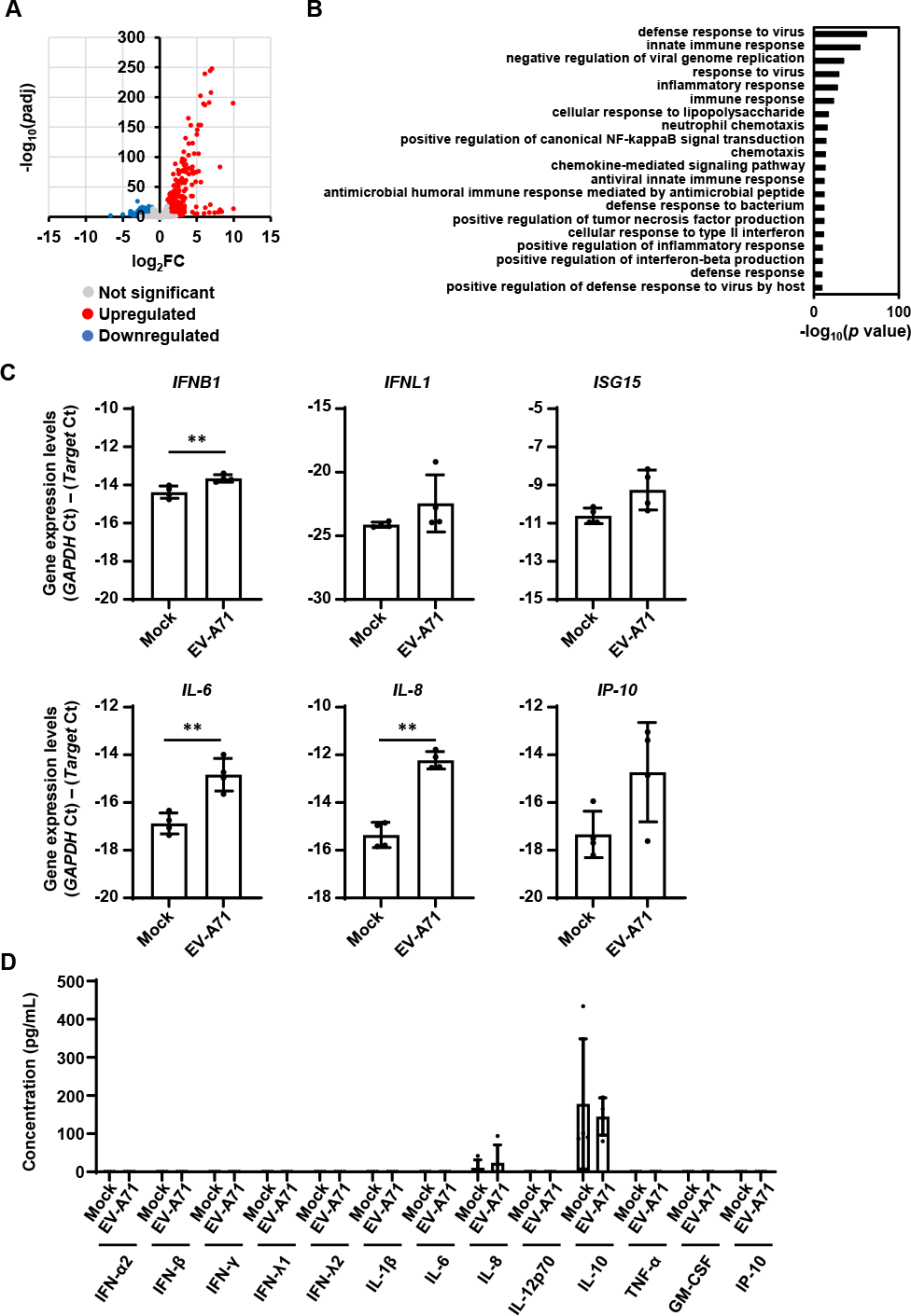
Innate immune response analysis in the EV-A71-infected intestinal MPS. The intestinal MPS was infected with EV-A71 at 0.142 TCID_50_/cell. (**A**) RNA-seq analysis was performed using the intestinal MPS infected at 7 dpi. A volcano plot of differentially expressed genes between mock- and EV-A71-infected intestinal MPS (log_2_ fold-change > 1, adjusted *P*-value [*P*adj] < 0.05). Blue and red dots represent genes that are downregulated and upregulated, respectively, by EV-A71 infection. (**B**) GO enrichment analysis of upregulated genes in EV-A71-infected cells compared with mock-infected cells. (**C**) Gene expression of *IFNB1*, *IFNL1*, *ISG15, IL-6, IL-8, or IP-10* in the intestinal MPS at 7 dpi was examined by RT-qPCR analysis. Unpaired two-tailed Student’s *t*-test (**P < 0.01). Data are represented as mean ± SD (*n* = 4). (**D**) The concentration of cytokines (IFN-α2, IFN-β, IFN-γ, IFN-λ1, IFN-λ2, IL-1β, IL-6, IL-8, IL-12p70, IL-10, TNF-α, GM-CSF, and IP-10 [CXCL10]) in the culture supernatant of the intestinal MPS at 7 dpi was evaluated using a bead-based multiplex immunoassay. Data are shown as mean ± SD (*n* = 4).

To investigate intestinal responses against viral infection, bulk RNA-sequencing (RNA-seq) was performed on the intestinal MPS infected with EV-A71 for 7 days. As shown in Fig. 2A, 418 gene expressions were upregulated, and 716 gene expressions were downregulated by EV-A71 infection. GO enrichment analysis was performed (Fig. 2B; Fig. S4A) and revealed that GO terms associated with “defense response to virus” or “innate immune response” were enriched in the genes upregulated by EV-A71 infection. RT-qPCR analysis also showed that the expression of *interferon beta 1* (*IFNB1*), *lambda 1* (*IFNL1*), *interferon-stimulated gene 15* (*ISG15*), *interleukin-6* (*IL-6*), *IL-8,* and *IP-10* was upregulated by EV-A71 infection (Fig. 2C). However, the Ct values of these genes in EV-A71-infected cells were high, indicating that their mRNA levels were low. The secretion of IFNs and ILs was not induced by EV-A71 infection (Fig. 2D). Because the intestinal MPS responds to other exogenous stimuli, such as lipopolysaccharide (LPS), a major component of the outer membrane of gram-negative bacteria, and TNF-α, at both the mRNA and protein levels (Fig. S4B and C), the low responsiveness of the intestinal MPS appears to be specific to EV-A71. These results suggest that the IFN signaling activated by EV-A71 infection was insufficient to induce cytokine production, and therefore, EV-A71 was not eliminated from the intestinal MPS.

### Intestinal epithelial responses to EV-A71 infection in the intestinal MPS

**Fig 3:**
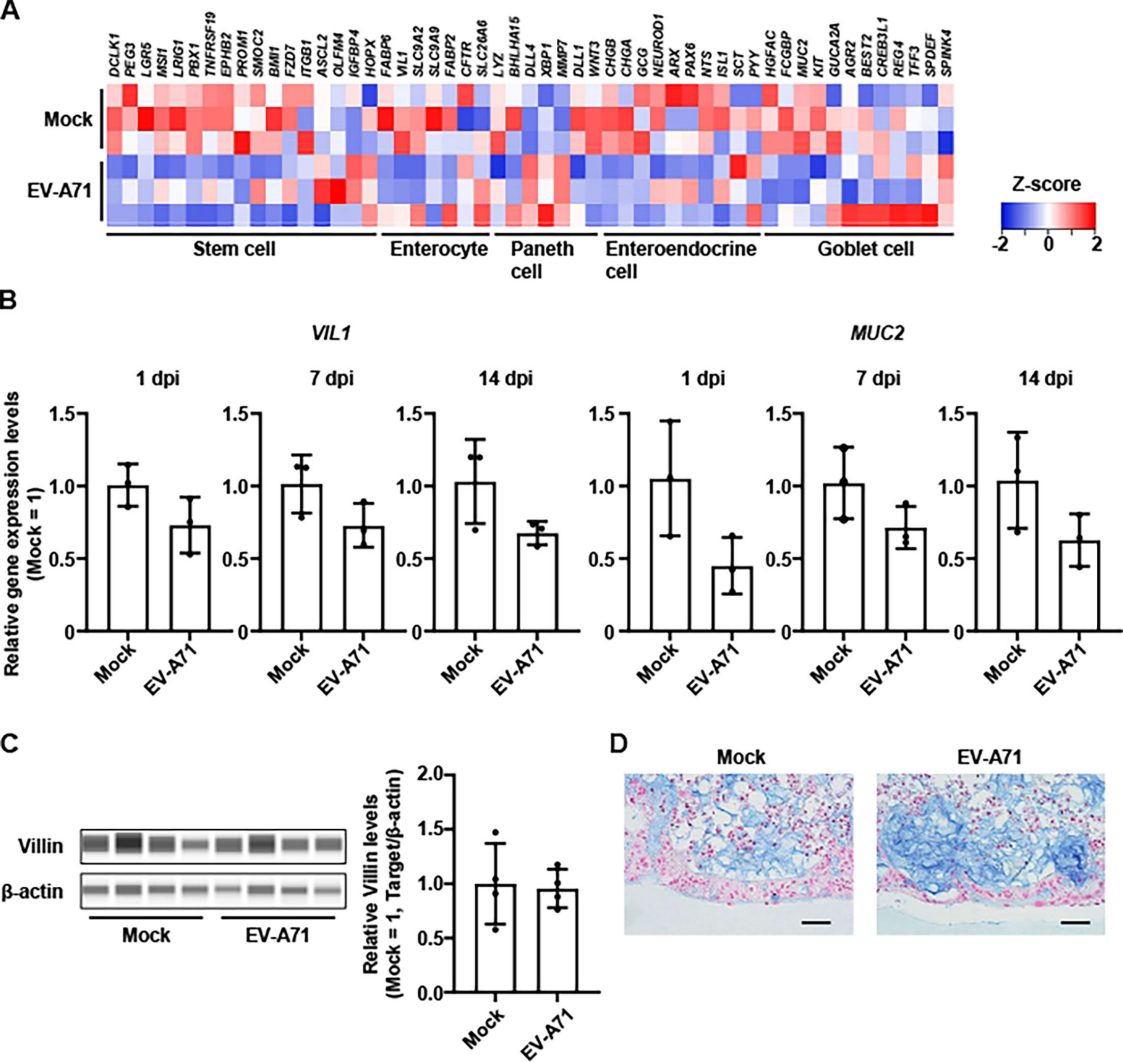
Analysis of intestinal epithelial cell markers in the EV-A71-infected intestinal MPS. The intestinal MPS was infected with EV-A71 at 0.142 TCID_50_/cell. (**A**) RNA-seq analysis was performed using the intestinal MPS at 7 dpi. Heatmap showing the gene expression level of intestinal epithelial cell markers in the intestinal MPS infected with EV-A71. (**B**) Gene expression of intestinal epithelial cell markers in the intestinal MPS was examined by RT-qPCR analysis. Gene expression in the mock was taken as 1.0. Data are represented as mean ± SD (*n* = 3). (**C**) Protein level of Villin in the intestinal MPS at 7 dpi as examined by capillary-based immunoassay. Protein level in the mock was taken as 1.0. Data are shown as mean ± SD (*n* = 4). (**D**) Alcian blue staining of the intestinal MPS at 4 dpi. Scale bars represent 50 µm.

The expression of intestinal epithelial markers in the EV-A71-infected intestinal MPS was evaluated to examine the impact of viral infection on epithelial cells. Heatmap indicated that EV-A71 infection did not affect the expression of various intestinal epithelial cell markers (Fig. 3A). Consistently, RT-qPCR analysis showed that the expression of enterocyte marker, *Villin 1* (*VIL1*), and goblet cell marker, *Mucin 2* (*MUC2*), was not significantly changed at 1, 7, and 14 dpi (Fig. 3B). Protein level of Villin remained unchanged by EV-A71 infection (Fig. 3C; Fig. S3). Alcian blue staining also revealed mucin secretion in both mock- and EV-A71-infected cells (Fig. 3D). Therefore, these findings suggest that while EV-A71 persistently infects the intestinal MPS, it does not cause intestinal epithelial damage.

### IFN-mediated inhibition of EV-A71 infection in the intestinal MPS

**Fig 4:**
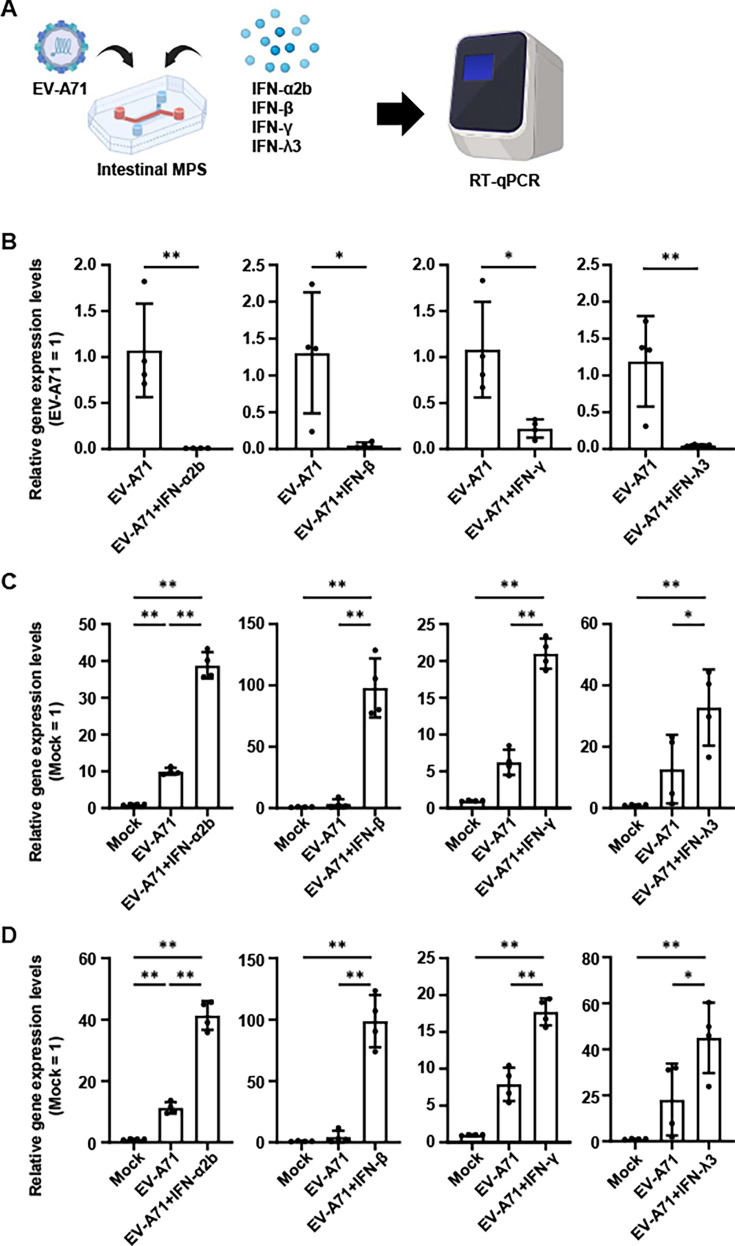
IFN treatment-mediated inhibition of EV-A71 infection in the intestinal MPS. (**A**) The intestinal MPS were treated with IFN-α2b, IFN-β, IFN-γ, or IFN-λ3 concurrently with EV-A71 infection at 0.142 TCID_50_/cell. (**B–D**) Relative expression of *EV-A71 VP1* (**B**), *ISG15* (**C**), or *MX1* (**D**) in the intestinal MPS at 7 dpi was examined by RT-qPCR analysis. Gene expression in the mock was taken as 1.0. Statistical significance was determined by an unpaired two-tailed Student’s *t*-test (*P < 0.05 and **P < 0.01) (**B**) and one-way ANOVA, followed by Tukey’s *post hoc* test (*P < 0.05 and **P < 0.01) (**C and D**). Data are represented as mean ± SD (*n* = 4).

It is known that EV-A71 evades the host innate immune response to promote viral replication (7). In Fig. 2D, EV-A71 infection did not induce the secretion of IFNs in the intestinal MPS. To determine whether the EV-A71 infection in the intestinal MPS can be suppressed by the IFN-mediated antiviral responses, we infected the intestinal MPS with EV-A71 and simultaneously treated it with type I IFNs (IFN-α2 and IFN-β), type II IFN (IFN-γ), or type III IFN (IFN-λ3) (Fig. 4A). At 7 dpi, the expression of viral mRNA and immune response-related genes was analyzed by RT-qPCR analysis. The expression of viral mRNA in the intestinal MPS was significantly decreased by treatment with all types of IFNs (Fig. 4B). The expression of *ISG15* (Fig. 4C) or *MX dynamin-like GTPase 1* (*MX1*) (Fig. 4D) was significantly higher in EV-A71-infected cells treated with IFNs than in cells infected with EV-A71 alone. These results demonstrate that EV-A71 infection in the intestinal MPS was inhibited by type I, II, and III IFN treatment.

## DISCUSSION

We recapitulated the intestinal responses to EV-A71 infection by using the intestinal MPS, whose characteristics resemble those of the human intestine. EV-A71 infected the intestinal MPS for 2 weeks without inducing significant cellular toxicity or cytokine secretion. Consistently, EV-A71 has been reported to persistently infect the human intestine for several weeks (8), with viral RNA detected in 48.1% of stool samples from HFMD patients even after 4 weeks of infection (5). However, the incidence of diarrhea in infected patients remained low (approximately 7%) (9). It is suggested that our intestinal MPS, persistently infected without substantial epithelial damage, could model the clinical features of EV-A71 infection.

EV-A71 continuously infected the intestinal MPS for 14 days; however, cytokine secretion, including IFNs, was not induced in the culture supernatant. The 3C and 2A proteases of EV-A71 are known to antagonize IFN signaling (10, 11). These viral proteases may have suppressed IFN signaling in the intestinal MPS. Moreover, IFN treatment inhibited EV-A71 infection in the intestinal MPS, suggesting that viral proteins suppress IFN signaling, thereby contributing to the long-term persistence of EV-A71 infection in this model.

There are currently no approved drugs for enterovirus infection. The antiviral activity of rupintrivir against human RV, a member of the genus *Enterovirus*, has been evaluated in clinical trials (12). Administration of rupintrivir, either prophylactically or therapeutically, reduced the number of patients positive for viral RNA but did not significantly decrease the incidence of clinical colds. These findings highlight the need to develop more effective drugs for viral infections. Because the intestinal MPS models can be used to evaluate drug efficacy, toxicity, and metabolism (13), they are expected to facilitate the development of antiviral drugs against EV-A71 infection.

While EV-A71 infection typically causes mild and self-limiting diseases, severe cases can lead to neurological complications (14). To prevent severe disease, it is crucial to reproduce how EV-A71 replicates in the intestine, transmits to the CNS, and causes neurological dysfunction and to elucidate the underlying mechanisms. Some studies have demonstrated that multiorgan interactions can be modeled by connecting different organ MPS models. For example, colon organoids have been integrated with visceral sensory ganglion organoids on a microfluidic device, successfully recapitulating the propagation of gut-derived amyloid and tau to the brain (15). In a similar way, it may be possible to model viral dissemination from the intestine to the CNS by connecting our intestinal MPS, persistently infected with EV-A71, with brain organoids using a microfluidic device. We hope that our study will provide new insight into the pathogenesis of EV-A71 infectious disease.

## MATERIALS AND METHODS

The methods for differentiation and analysis of intestinal MPS are described in detail in reference 4; here, we provide a summary for the reader.

### Cell culture

Human ES cells, KhES-3 (provided by Kyoto University), were maintained on 0.5 μg/cm^2^ recombinant human laminin 511 E8 fragments (i-Matrix-511 silk; Cat# 892021; Nippi) with StemFit AK02N medium (Cat# RCAK02N; Ajinomoto Healthy Supply). Cells were passaged every 6 days by treating cell colonies with TrypLE Select Enzyme (Cat# 12563011; Thermo Fisher Scientific) for 10 min at 37°C and seeded with StemFit AK02N medium containing 10 μM Y-27632 (Cat# 034-24024; FUJIFILM Wako Pure Chemical). Human ES cells were also maintained on Matrigel hESC-Qualified Matrix (Cat# 354277, Corning) with mTeSR1-cGMP medium (Cat# ST-85850, STEMCELL Technologies). For passaging, human ES cells were treated with 1 mg/mL Dispase II (Cat# 04942078001, Roche) for 9 min at 37°C. Human ES cells were used following the Guidelines for Derivation and Utilization of Human Embryonic Stem Cells of the Ministry of Education, Culture, Sports, Science and Technology of Japan.

RD cells (Cat# JCRB9072) were obtained from the JCRB Cell Bank and cultured with DMEM (Cat# 044-29765; FUJIFILM Wako Pure Chemical) supplemented with 10% fetal bovine serum, 1× GlutaMAX, 1× non-essential amino acids (NEAAs) (Cat# 11140-050; Thermo Fisher Scientific), and 1× penicillin-streptomycin.

### Intestinal MPS

Human ES cells were seeded on Matrigel Growth Factor Reduced Basement Membrane (Cat# 354230; Corning)- or Cultrex UltiMatrix Reduced Growth Factor Basement Membrane Extract (Cat# BME001-10; R&D Systems)-coated cell culture plates and cultured with StemFit AK02N medium or mTeSR1-cGMP medium containing 10 μM Y-27632 for 1 day. Cells were treated with 100 ng/mL Activin A (Cat# 338-AC; R&D Systems) in RPMI 1640 medium (Cat# R8758-500; Sigma-Aldrich) supplemented with 1× B-27 Supplement Minus Vitamin A (Cat# 12587001; Thermo Fisher Scientific), 1× GlutaMAX (Cat# 35050-079; Thermo Fisher Scientific), and 1× penicillin-streptomycin for 3 days. Next, cells were dissociated and resuspended at 5 × 10^6^ cells/mL in the intestinal differentiation medium (Advanced DMEM/F12 [Cat# 12634028; Thermo Fisher Scientific] supplemented with 2% FBS, 1× GlutaMAX, and 1× penicillin-streptomycin) containing 100 ng/mL fibroblast growth factor 2 (FGF2) (Cat# 160-0010-3; Katayama Chemical Industries) and 10 μM Y-27632. Then, a cell suspension (10 µL) was injected into the Matrigel- or Cultrex-coated top channel of the PDMS device. After 1 h, the intestinal differentiation medium, containing 100 ng/mL FGF2 and 10 μM Y-27632, was added to the top channel of the PDMS device. During days 5–7, cells were maintained with an intestinal differentiation medium containing 100 ng/mL FGF2. From day 8 to 12, cells were maintained with a 3:1 mixture of the intestinal differentiation medium and Wnt family member 3A (WNT3A)-, RSPO3-, and noggin (NOG)-conditioned medium supplemented with 50 ng/mL epidermal growth factor (EGF) (Cat# AF-100-15; PeproTech) and 20 ng/mL insulin-like growth factor 1 (Cat# AF-100-11; PeproTech). WNT3A, RSPO3, and NOG-conditioned medium was prepared using L-WRN cells (Cat# CRL-3276; American Type Culture Collection [ATCC]). During days 13–24, cells were maintained with a 1:1 mixture of the intestinal differentiation medium containing 1% NEAA (Cat# 11140-050; Thermo Fisher Scientific), 1× B-27 Supplement Minus Vitamin A, and 1× N2 supplement (Cat# 141-08941; FUJIFILM Wako Pure Chemical), and Hepatocyte Culture Media BulletKit (Cat# CC-3198; LONZA) supplemented with 10 ng/mL EGF, 15 µM forskolin (Cat# 063-02193; FUJIFILM Wako Pure Chemical), 10 µM PD98059 (Cat# S1177; Selleck Chemicals), 2.5 µM 5-aza-2′-deoxycytidine (Cat# 018-20941; FUJIFILM Wako Pure Chemical), 0.25 µM A-83-01 (Cat# 035-24113; FUJIFILM Wako Pure Chemical), 2.5 µM (2ʹZ,3ʹE)-6-Bromoindirubin-3ʹ-oxime (Cat# 361550; Sigma-Aldrich), and 5 µM (3,5-Difluorophenylacetyl)-Ala-Phg-OBu^t^ (Cat# 3219-v; Peptide Institute). Medium flow was maintained using a Mini Infinity Rocker (Next Advance) at a flow rate of 0.1 cycle/min or using an OrganoFlow (Mimetas) programmed to cycle every 4 min to a maximum angle of 25° during days 7–24.

To plate the intestinal MPS onto a multi-well plate, the human ES cell-derived intestinal tissue was recovered from the microfluidic devices by separating the top and bottom PDMS layers. The collected intestinal tissue was then replated onto the Matrigel- or Cultrex-coated wells.

### Microfluidic devices

The microfluidic device consists of two layers of microchannels separated by a semipermeable membrane (16). The microchannel layers were fabricated from polydimethylsiloxane (PDMS) using a soft lithography method (17). PDMS prepolymer (Cat# Sylgard 184; Dow Corning) at a ratio of 10:1 (base to curing agent) was cast against a mold composed of SU-8 2150 (MicroChem) patterns formed on a silicon wafer. The cross-sectional size of the microchannels was 1 mm in width and approximately 300 μm in height. To introduce solutions into the microchannels, access holes were punched through the PDMS using a 6-mm biopsy punch (Kai Corporation). Two PDMS layers were bonded to a semipermeable polyethylene terephthalate (PET) membrane containing 3.0 μm pores (Cat# 353091; Corning) using a thin layer of liquid PDMS prepolymer as the mortar (18). PDMS prepolymer was spin-coated (4,000 rpm for 60 s) onto a glass slide. Subsequently, both the top and bottom channel layers were placed on a glass slide to transfer the thin layer of PDMS prepolymer onto the embossed PDMS surfaces. The PET membrane was placed on the top layer and bonded to the bottom layer. The combined layers were left at room temperature for 1 day to remove air bubbles and then put into an oven at 60°C overnight to cure the PDMS adhesive. PDMS-based microfluidic devices (PDMS devices) were sterilized by placing them under ultraviolet light for 1 h before use.

### EV-A71 infection

EV-A71 (Cat# VR-1432) was obtained from ATCC. EV-A71 was propagated in RD cells. To prepare virus stocks, the culture medium of RD cells was replaced with DMEM supplemented with 2% fetal bovine serum, 1× GlutaMAX, 1× NEAA, and 1× penicillin-streptomycin, and the following day, the cells were infected with EV-A71. Culture supernatants were collected at 3 dpi and stored at −80°C until use.

For EV-A71 infection, the intestinal MPS was placed on a multi-well plate. The intestinal MPS (0.142 TCID_50_/cell) or RD cells (0.1 MOI) were infected with EV-A71. At 1 dpi, the cells were washed twice with PBS, and the medium was replaced with fresh medium. For rupintrivir treatment, the cells were treated with 1 µM rupintrivir (Cat# HY-106161; MedChemExpress) at the time of infection (Fig. 1I) or 1 dpi (Fig. 1J). For recombinant IFN treatment, the cells were treated with 10 ng/mL IFN-α2b (Cat# HZ-1072; ProteinTech), IFN-β (Cat# 300-02BC; PeproTech), IFN-γ (Cat# 300-02; PeproTech), or IFN-λ3 (Cat# 5259-IL-025; R&D Systems) at the time of infection.

### Viral titration

Viral titers were measured using a median tissue culture infectious dose (TCID_50_) assay. RD cells were seeded into 96-well cell culture plates (Cat# 167008; Thermo Fisher Scientific). Samples were serially diluted 10-fold from 10^−1^ to 10^−8^ in cell culture medium, transferred onto the cells, and incubated at 37°C for 72 h. Cytopathic effects were evaluated under a microscope. TCID_50_/mL was calculated using the Reed-Muench method.

### TNF-α and LPS treatment

The intestinal MPS was treated with 30 ng/mL TNF-α (Cat# 300-01A; PeproTech) and 1 µg/mL LPS (Cat# L2654-1MG; Sigma-Aldrich) for 8 days. The cell culture supernatant was collected from the top and bottom channels of the microfluidic devices separately.

### RT-qPCR

Total RNA was isolated using ISOGENE (Cat# 319-90211; Nippon Gene). cDNA was synthesized with the Superscript VILO cDNA Synthesis Kit (Cat# 11754250; Thermo Fisher Scientific). RT-qPCR was performed with SYBR Green PCR Master Mix (Cat# 4385614; Thermo Fisher Scientific) using a StepOnePlus real-time PCR system, QuantStudio 1, or QuantStudio 3 Real-Time PCR System (Thermo Fisher Scientific). In Fig. 1J, 3B and 4B through D, the relative quantification of target mRNA levels was performed using the 2^-ΔΔCt^ method. The values were normalized to the housekeeping gene *glyceraldehyde 3-phosphate dehydrogenase* (*GAPDH*). In Fig. 2C and Fig. S4B, the difference between the Ct value of *GAPDH* and that of the target gene is shown on the *y*-axis. Primer sequences are summarized in Table S1.

### WST-8 assay

Cell viability was assessed by Cell Counting Kit-8 (Cat# 343-07623, Dojindo) according to the manufacturer’s protocol. The viability of mock-infected cells was taken as 100%.

### Immunofluorescence staining of paraffin sections

For immunofluorescence staining of paraffin sections (Fig. S1A and B), the intestinal MPS was fixed with 4% paraformaldehyde for 15 min, harvested, and used to prepare paraffin sections. Paraffin-embedded tissue sectioning was performed by the Applied Medical Research Laboratory. Sections were deparaffinized with xylene and rehydrated through a graded ethanol series. For antigen retrieval, sections were processed by heating at 90°C in HistoVT One (Cat# 06380-05; Nacalai Tesque) for 20 min. Sections were permeabilized using Tris-buffered saline with 0.1% Tween 20 Detergent (Cat# 12750-81; Nacalai Tesque), blocked with Blocking One Histo (Cat# 06349-64; Nacalai Tesque) for 10 min, and incubated in Tris-buffered saline with 0.1% Tween 20 Detergent with primary antibodies at 4°C overnight. They were washed with PBS and incubated in Tris-buffered saline with 0.1% Tween 20 Detergent containing Alexa Fluor 488- or 594-conjugated secondary antibodies for 45 min at room temperature. Sections were finally washed and mounted with DAPI Solution (Cat# 19178-91; Nacalai Tesque) and analyzed using a FV3000 confocal microscope (Evident). Antibodies, including a previously reported in-house anti-EV-A71 antibody (19), are summarized in Table S2.

### Immunofluorescence staining of frozen sections

For immunofluorescence staining of frozen sections (Fig. 1C), the intestinal MPS was fixed with 4% paraformaldehyde (Cat# 163-20145; FUJIFILM Wako Pure Chemical) for 15 min, harvested, and used to prepare frozen sections (5 µm). For antigen retrieval, sections were processed by heating at 70°C in HistoVT One (Cat# 06380-05; Nacalai Tesque) for 20 min. Sections were permeabilized using Tris-buffered saline with 0.1% Tween 20 Detergent (Cat# 12750-81; Nacalai Tesque), blocked with SuperBlock Blocking Buffer (Cat# 37580; Thermo Fisher Scientific) for 10 min, and incubated in Tris-buffered saline with 0.1% Tween 20 Detergent with primary antibodies for 2 h at room temperature. They were washed with PBS and incubated in Tris-buffered saline with 0.1% Tween 20 Detergent containing Alexa Fluor 488- or 594-conjugated secondary antibodies for 45 min at room temperature. Sections were finally washed and mounted with DAPI Solution (Cat# 19178-91; Nacalai Tesque) and analyzed using a BZ-X700 fluorescence microscope (Keyence Corporation). Antibodies are summarized in Table S2.

### Hematoxylin and eosin and Alcian blue staining

The intestinal MPS was fixed with 4% paraformaldehyde for 15 min, harvested, and used to prepare paraffin sections. Paraffin-embedded tissue sectioning and hematoxylin and eosin and Alcian blue staining were performed by the Applied Medical Research Laboratory.

### Capillary-based immunoassay

The intestinal MPS was lysed in RIPA buffer (Cat# 89900; Thermo Fisher Scientific) containing a Halt Protease Inhibitor Cocktail (Cat# 78438; Thermo Fisher Scientific). After the centrifugation, the supernatants were collected. Total protein from a human adult normal colon was purchased from BioChain Institute Inc. (Cat# P1234090). Antibody-based protein quantification was performed using a Jess system (ProteinSimple) with a 12–230 kDa Separation Module (Cat# SM-W001; ProteinSimple) as instructed. Antibodies used for this analysis are summarized in Table S2. Data were analyzed and visualized using Compass for Simple Western software (ProteinSimple). Jess protein images are shown throughout the manuscript.

### RNA-seq analysis

RNA was isolated from the EV-A71-infected intestinal MPS. RNA integrity was assessed with a 2100 Bioanalyzer (Agilent Technologies). RNA-seq libraries were constructed using an Illumina Stranded mRNA Prep, Ligation Kit (Illumina, Cat#20040532) according to the manufacturer’s instructions. Sequencing was performed on an Illumina NextSeq 2000 with the paired-end mode. FASTQ files were generated using bcl2fastq-2.20. Adapter sequences and low-quality bases were trimmed from the raw reads using cutadapt version 4.6 (20). Trimmed reads were mapped to human reference genome sequences (hg38) using STAR version 2.7.11a (21) with GENCODE (release 32, GRCh38.p13) (22) GTF file. Uniquely and properly mapped reads were used for further analysis. Raw read counts were calculated using htseq-count version 2.0.5 (23) with the GENCODE GTF file. Differential expression analysis was performed by DESeq2 version 1.42.0 (24) using the Wald test. *P* values were corrected for multiple testing using the Benjamini and Hochberg method and represented as the false discovery rate. The accession number for RNA-seq data reported in this study is GSE310224.

### Bead-based multiplex immunoassay

Cytokine and chemokine concentrations in the culture supernatants of EV-A71-infected intestinal MPS were measured using a LEGENDplex Human Anti-Virus Response Panel (Cat# 740390; BioLegend). Flow cytometry was performed according to the manufacturer’s instructions using a MACSQuant Analyzer 10 (Miltenyi Biotec).

### Statistical analysis

Statistical significance was evaluated using an unpaired two-tailed Student’s *t*-test or one-way analysis of variance, followed by Tukey’s *post hoc* test. Data are representative of three independent experiments. Details are described in the figure legends.

## Data Availability

The accession number for RNA-seq data reported in this study is GSE310224. Information required to reanalyze the data reported in this paper is available from the lead contact upon request.

## SUPPLEMENTAL MATERIAL

Supplemental material jvi.00250-26-s0001.pdf Figures S1 to S4; Tables S1 and S2.

ASM does not own the copyrights to Supplemental Material that may be linked to, or accessed through, an article. The authors have granted ASM a non-exclusive, world-wide license to publish the Supplemental Material files. Please contact the corresponding author directly for reuse.
