# Supplementary material for: Modeling human enterovirus A71 infection using an intestinal microphysiological system
Source: J Virol. 2026 Apr 21;100(5):e00250-26. doi: 10.1128/jvi.00250-26 (PMC13185576; doi:10.1128/jvi.00250-26)
Supplement: Supplemental material — Figures S1 to S4; Tables S1 and S2. [file jvi.00250-26-s0001.pdf]

Supplementary figure legends

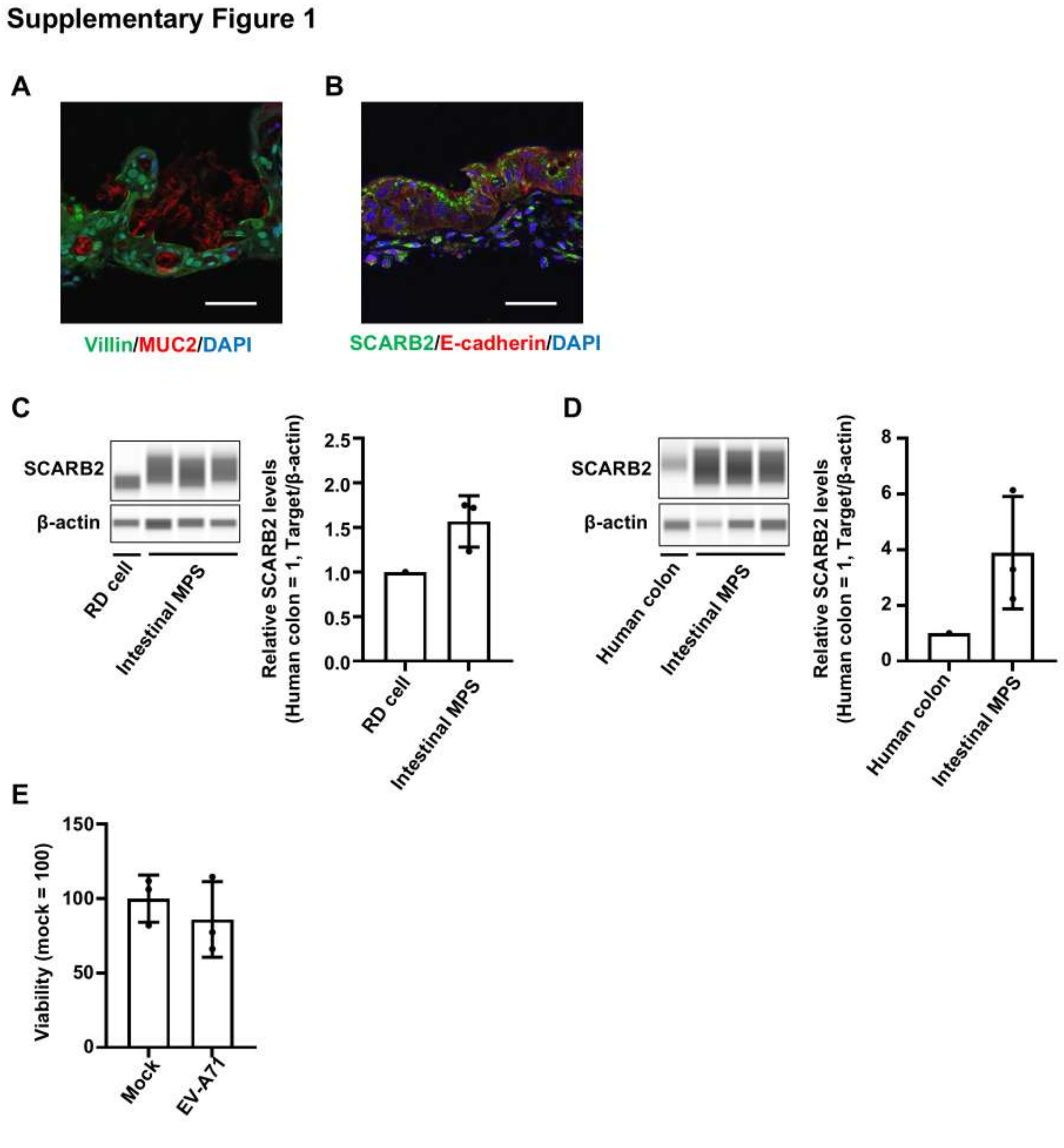

Figure S1. EV-A71 infection using the intestinal MPS

(**A**) Immunofluorescent staining of intestinal epithelial cell marker (Villin, green) and goblet cell marker (MUC2, red) in the intestinal MPS. Nuclei were counterstained with DAPI (blue). Scale bar represents 50 µm. (**B**) Immunofluorescent staining of SCARB2 (green) and intestinal epithelial cell marker (E-cadherin, red) in the intestinal MPS. Nuclei were counterstained with DAPI (blue). Scale bar represents 50 µm. (**C**-**D**) Protein level of SCARB2 in RD cells (**C**), human colon (**D**), and the intestinal MPS was examined by capillary-based immunoassay. Data are represented as mean ± SD (n = 1 for RD cells and human colon, n = 3 for the intestinal MPS). (**E**) WST-8 assays of mock- or EV-A71-

infected intestinal MPS. Data are represented as mean ± SD (n = 3).

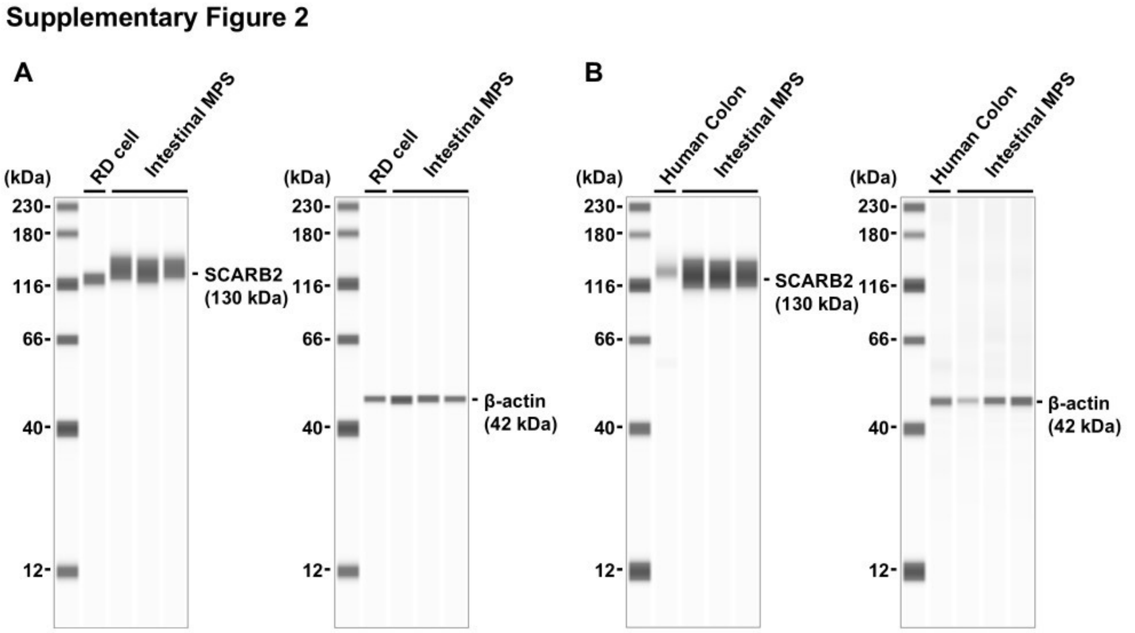

Figure S2. Protein expression analysis in the intestinal MPS

(**A**) Full gel images of capillary-based immunoassay of **Figure S1C**. (**B**) Full gel images of capillary-based immunoassay of **Figure S1D**.

Supplementary Figure 3

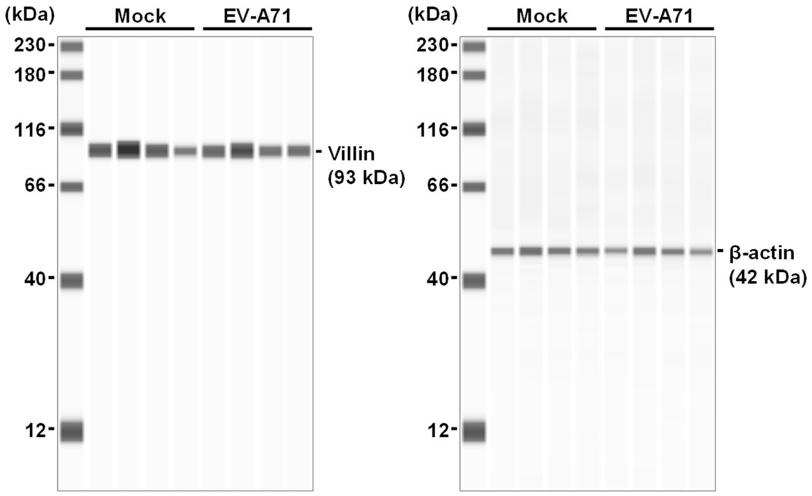

Figure S3. Protein expression analysis in EV-A71-infected intestinal MPS

Full gel images of capillary-based immunoassay of **Figure 3C**.

Supplementary Figure 4

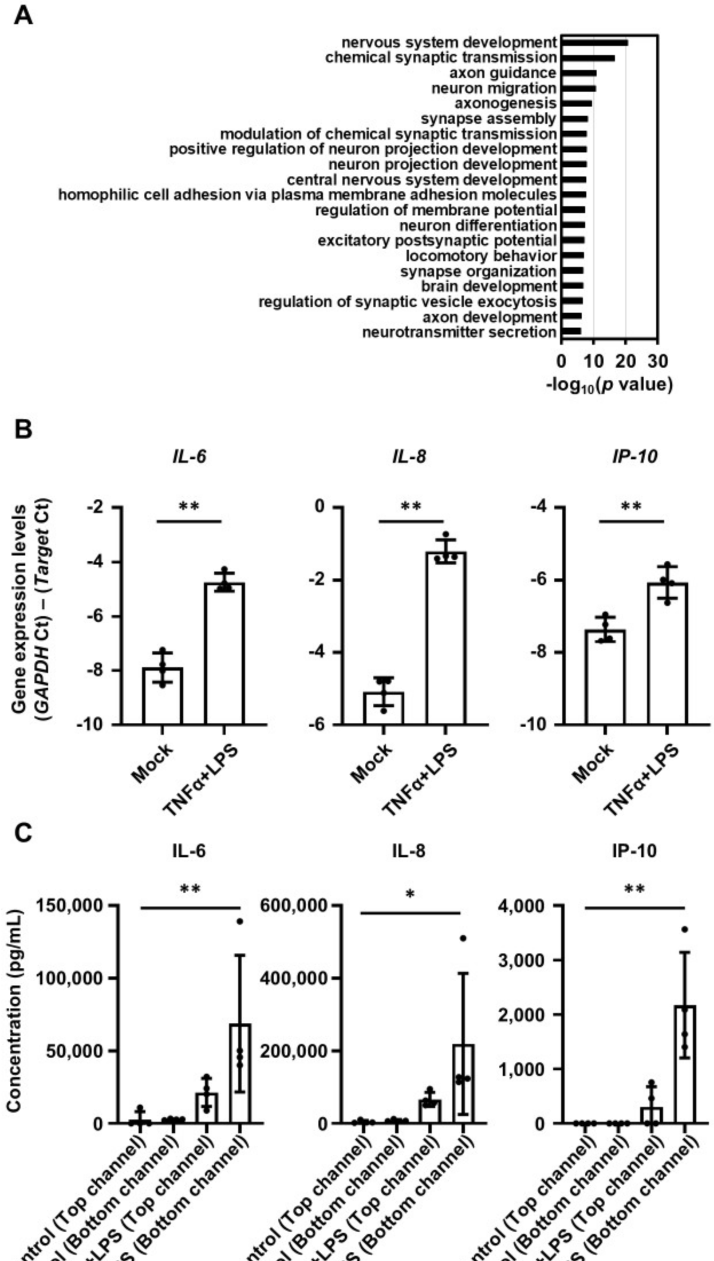

Figure S4. Innate immune response analysis in the EV-A71-infected intestinal MPS or intestinal MPS treated with other exogenous stimuli

(**A**) The intestinal MPS was infected with EV-A71 at 0.142 TCID50/cell. GO enrichment analysis of downregulated genes in EV-A71-infected cells compared with mock-infected cells. (**B-C**) The intestinal MPS was treated with 30 ng/mL TNF-α and 1

μg/mL lipopolysaccharide (LPS) for 8 days. (**B**) Gene expressions of *IL-6, IL-8,* and *IP-10* in the intestinal MPS after TNF-α and LPS treatment were examined by RT-qPCR analysis. Unpaired two-tailed Student's *t*-test (**p<0.01). Data are represented as mean ± SD (n = 4). (**C**) The concentration of cytokines (IL-6, IL-8, and IP-10 [CXCL10]) in the culture supernatant of the intestinal MPS after TNF-α and LPS treatment was evaluated using a bead-based multiplex immunoassay. One-way ANOVA, followed by Dunnett's post hoc test (*p<0.05, **p<0.01). Data are shown as means ± SD (n = 4).

Supplementary tables

Table S1. Primers used for RT-qPCR

Gene	Forward primers	Reverse primers
GAPDH	GGAGCGAGATCCCTCCAAAAT	GGCTGTTGTCATACTTCTCATGG
EV-A71 VP1	GGAGATAGGGTGGCAGATG	CCAATTTCAGCGGCTTGGAG
VIL1	CTGAGCGCCCAAGTCAAAG	AGCAGTCACCATCGAAGAAGC
MUC2	GAGGGCAGAACCCGAAACC	GGCGAAGTTGTAGTCGCAGAG
IFNB1	ATGACCAACAAGTGTCTCCTCC	GGAATCCAAGCAAGTTGTAGCTC
IFNL1	CACATTGGCAGGTTCAAATCTCT	CCAGCGGACTCCTTTTTGG
IL-6	ACTCACCTCTTCAGAACGAATTG	CCATCTTTGGAAGGTTCAGGTTG
IL-8	TTTTGCCAAGGAGTGCTAAAGA	AACCCTCTGCACCCAGTTTTC
IP-10	GTGGCATTCAAGGAGTACCTC	TGATGGCCTTCGATTCTGGATT
ISG15	CGCAGATCACCCAGAAGATCG	TTCGTCGCATTTGTCCACCA
MX1	CTTATCCGTTAGCCGTGGTG	CAAGGTGGAGCGATTCTGAG

Table S2. Antibodies used for immunofluorescence staining and Jess analysis

Antigen	Host	Company	Catalogue
E-cadherin	Mouse	Santa Cruz Biotechnology	sc-8426
Villin	Mouse	Santa Cruz Biotechnology	sc-58897
MUC2	Mouse	Santa Cruz Biotechnology	sc-515032
EV-A71 VP1	Rabbit	In-house (1)	
dsRNA	Mouse	SCICONS	10010200
SCARB2	Rabbit	Proteintech	27102-1-AP
β-actin	Mouse	Sigma-Aldrich	A5441
Alexa 488-conjugated anti-	Donkey	Thermo Fisher Scientific	A21206

mouse IgG antibody			
Alexa 594-conjugated anti-rabbit IgG antibody	Goat	Thermo Fisher Scientific	A11032

Supplementary reference

1. Kotani O, Iwata-Yoshikawa N, Suzuki T, Sato Y, Nakajima N, Koike S, Iwasaki T, Sata T, Yamashita T, Minagawa H. 2015. Establishment of a panel of in-house polyclonal antibodies for the diagnosis of enterovirus infections. Neuropathology 35:107-121.
